# How factors of the therapeutic alliance interact with oxytocin neurotransmission in psychotherapy

**DOI:** 10.3389/fpsyt.2025.1659841

**Published:** 2025-12-11

**Authors:** Marcus Eckert, Eva Schandro

**Affiliations:** Apollon University of Applied Sciences, Bremen, Germany

**Keywords:** oxytocin, therapeutic alliance, attachment, empathy, social synchrony, cooperation, social support

## Abstract

Oxytocin (OT) neurotransmission has emerged as a promising target for alleviating psychiatric symptoms associated with depression, stress, and fear. This mini-review focuses on the therapeutic alliance, highlighting five core components: (1) attachment, (2) empathy, (3) social synchrony, (4) trust and cooperation, and (5) social support. We explore how these factors both influence and are modulated by the oxytocinergic system. Based on current empirical evidence, we propose a conceptual framework in which OT-mediated mechanisms dynamically and reciprocally strengthen the therapeutic alliance. We postulate that activation of the OT system by one or more of these components may synergistically enhance all alliance factors, creating a self-reinforcing cycle with potential therapeutic benefits. Finally, we discuss the clinical implications of this model and identify key avenues for future research.

## Introduction

Oxytocin (OT) is a neuropeptide closely associated with both physical and mental health. Numerous studies indicate that OT can reduce stress and anxiety while promoting resilience and psychological well-being (1, 2). Moreover, OT has been implicated in various psychiatric disorders, including social anxiety disorder, affective disorders, autism spectrum disorder, schizophrenia, post-traumatic stress disorder, and borderline personality disorder (3). It is important to note, however, that findings vary depending on the methods used to measure OT—such as peripheral blood levels, central nervous system activity, or effects of exogenous OT administration—making direct comparisons challenging.

Due to these multifaceted associations, OT has increasingly gained attention as a potential therapeutic target in psychiatric treatment (2). Nevertheless, evidence regarding the therapeutic efficacy of OT-related interventions remains heterogeneous and sometimes contradictory, likely reflecting the influence of moderating variables such as individual differences, sex, and social context (4). Furthermore, methodological challenges persist, especially regarding the indirect nature of peripheral OT measurements as proxies for central oxytocinergic activity. Young (5) underscores the need for alternative pharmacological strategies to enhance OT neurotransmission, highlighting its therapeutic potential. Complementing this perspective, the present article focuses on psychological approaches to augmenting OT activity. Specifically, we emphasize the therapeutic alliance—a relational factor empirically linked to oxytocinergic processes (6).

It is crucial to acknowledge the considerable individual variability in OT effects, and that the relationships presented here provide only a preliminary overview that cannot fully capture the complexity and occasional inconsistencies in the literature. Nonetheless, we aim to introduce a conceptual model identifying five core factors empirically associated with both therapeutic alliance quality and OT activity: (1) attachment, (2) empathy, (3) social synchrony, (4) trust and cooperation, and (5) social support. First, while adult attachment theory does not map directly onto the therapeutic alliance, emerging evidence suggests that an attachment-based framework—particularly when considering oxytocin as a process marker—may offer valuable insights into relational dynamics in psychotherapy (7). Second, empathy, a widely recognized non-specific factor in psychotherapy outcomes (8), has a documented but context-dependent relationship with oxytocin (9). Recent research points to links between OT, behavioral synchrony, and even inter-brain synchrony during empathic engagement (10). Third, mutual trust and collaboration—core elements of the therapeutic relationship—have been associated with improved metacognitive functioning (11) and show dynamic, though sometimes inconsistent, associations with oxytocin (12). Finally, although often considered extra-therapeutic, social support has demonstrated bidirectional interactions with the therapeutic alliance (13) and is increasingly recognized as both a correlate and modulator of oxytocin-related processes (14, 15).

In the debate about the most suitable psychotherapy, Wampold (16) suggested non-specific factors such as the therapeutic alliance. The significance of the therapeutic alliance for psychotherapy outcomes has been robustly demonstrated—for example, Tschuschke et al. (17) report that alliance quality predicts treatment success more strongly than specific therapeutic modalities. Against this backdrop, OT has been proposed as a neurobiological substrate contributing to therapeutic effectiveness. A recent mini-review by Durante et al. (7) synthesized studies examining natural fluctuations and synchronization of OT levels between therapists and patients, finding preliminary evidence that OT covariation may be associated with treatment outcomes in major depressive disorder. However, this evidence is based on a limited number of studies, with no experimental or replication studies to date, highlighting the need for further research.

Studies employing exogenous OT administration have yielded mixed results. For instance, Grossman-Giron et al. (18) observed that intranasal OT improved concordance between patient and therapist perceptions of the alliance, while Ellenbogen et al. (19) reported that OT administration enhanced the therapeutic alliance particularly during early treatment phases. These findings suggest a potential for OT to strengthen the therapeutic relationship but also emphasize the necessity for more rigorous and replicable investigations.

In summary, this mini-review seeks to elucidate potential mechanisms through which OT may influence the quality of the therapeutic alliance. The hormonal context can generally be understood as a mediating factor in many psychological and social processes. This also applies to OT, of course. Studies involving exogenous OT administration often show its influence on behavior and experience. In addition, endogenous OT varies systematically with certain experiences and behaviors, often depending on the social context. This review postulates interactions between the factors of therapeutic alliance and OT on the one hand. On the other hand, the effect of OT on therapy outcomes is examined. In this analysis, OT emerges as a mediator of the therapeutic alliance’s effects on therapy outcomes. Furthermore, this review assumes reciprocal self-reinforcing processes whereby activation of the OT system promotes psychological processes that in turn make activation of the OT system more likely. We will also postulate spillover effects between the factors, which, however, must be confirmed by future research and which may offer valuable approaches for therapeutic action. In this sense, this conceptual framework provides a basis for future empirical studies and theoretical refinements.

## Attachment theory

Attachment theory, initially formulated by Bowlby and Ainsworth, distinguishes three primary attachment styles: secure, anxious, and avoidant (20, 21). Secure attachment is generally linked to better mental health outcomes, whereas insecure styles—anxious and avoidant—are risk factors for psychopathology. Oxytocin (OT) has been implicated as a multifaceted neurobiological correlate of attachment, influencing social behaviors and emotional regulation in ways moderated by individual attachment styles (22).

Empirical findings reveal complex, sometimes contradictory interactions between attachment and OT. For example, Nawa et al. (23) observed that playful mother–child interactions following traumatic stress increased maternal OT, which was inversely related to maternal distress and predicted reductions in children’s behavioral problems two years later. Bosmans et al. (24) proposed an attachment learning model emphasizing how supportive caregiving buffers stress responses via OT- and dopamine-mediated neurobiological reinforcement, promoting future attachment-seeking behavior.

Parental caregiving behavior itself appears to be reinforced through OT activation (25), although OT’s social-cognitive effects vary by attachment style. Bartz et al. (22) and Fang et al. (26) demonstrated that intranasal OT administration enhances perceptions of care and cooperation only in individuals with low attachment anxiety or avoidance, respectively, while those with high attachment insecurity exhibit attenuated or no effects. Rockliff et al. (27) further showed that secure attachment facilitates engagement in compassion-focused imagery after OT administration, an effect diminished in less securely attached individuals.

Conversely, OT may also promote more secure attachment representations. Zhang et al. (28) meta-analysis suggests that intranasal OT reduces behaviors associated with attachment insecurity in ambiguous social contexts. Similarly, Buchheim et al. (29) found that OT administration increased attachment security experiences among insecurely attached adults. In this regard, it is worth noting that contemporary attachment research focuses on the dimensional nature of attachment. For instance, Fraley et al. (30) suggest attachment to be best described as a continuous variable. A taxometric analysis showed that both parental and romantic relationships show signs of dimensionality.

Regarding psychotherapy, attachment styles can shift through the therapeutic process, with anxiously attached patients benefiting particularly from a strong therapeutic alliance (31, 32). Therapists can function as attachment figures, potentially activating patients’ OT systems. Manvelian et al. (33) reported that pairing insecurely attached students with warm, securely attached mentors trained in Emotionally Focused Therapy increased mentees’ attachment security, possibly mediated by OT activation. However, Karl et al. (34) found that while secure attachment priming reduced physiological arousal, OT administration did not, suggesting OT-independent pathways for increasing attachment security.

Finally, the therapeutic alliance, attachment security, and OT are theoretically intertwined. Several reviews highlight OT as a potential biomarker of the alliance (7, 35), and empirical evidence supports associations between attachment security and alliance quality (36). Nonetheless, these links remain preliminary and require rigorous longitudinal and mechanistic research to clarify their interplay.

In sum, OT’s role in attachment is complex and moderated by individual differences in attachment style. Behavioral interventions aiming to enhance OT should consider these nuances and ideally incorporate strategies to foster secure attachment representations, thereby optimizing both neurobiological and relational outcomes.

## Empathy

Empathy is widely recognized as a crucial factor influencing therapeutic alliance (37). Early work by Barraza and Zak (38) showed that inducing empathy via emotional video clips led to measurable increases in plasma OT, with greater empathic responses associated with larger OT changes. Building on this, Zak et al. (39) used similar stimuli in a quasi-experimental design, lacking a control group, limiting causal inferences. They reported age as a moderator, with older participants showing greater OT increases, and positive correlations between OT changes and empathic concern. Procyshyn et al. (40) also induced empathy through a video depicting a gravely ill child, observing a significant 14% rise in salivary OT among 173 healthy adults. Notably, individuals with a cognitive style biased toward empathizing, as conceptualized by Baron-Cohen and Wheelwright (41) empathizing–systemizing theory, showed more pronounced OT increases compared to those with systemizing biases (42). This bias is rooted in Baron-Cohen’s Empathizing–Systemizing (E-S) theory (41), which postulates two distinct cognitive dimensions—Empathizing and Systemizing—that are distributed continuously along a spectrum and associated with autism spectrum conditions. This means that individuals can exhibit different levels of each ability, with some people showing a strong preference for empathy, others for systematization, and still others a balance of both abilities. According to this theory, individuals high in empathizing are particularly attuned to the thoughts and emotions of others and possess a strong capacity to understand and respond appropriately to them. In contrast, individuals high in systemizing show a marked interest in, and aptitude for, analyzing and constructing technical, mechanical, or abstract rule-based systems (42). These findings suggest that individual differences modulate OT responses to empathy induction, highlighting the need to consider trait variability in therapeutic applications.

With regard to the therapeutic alliance, Fisher et al. (43) showed that therapists’ OT levels increased in response to patients’ negative emotions, thereby contributing to a reduction in patients’ depressive symptoms. This finding supports the assumption of an interaction in OT dynamics within clinical interactions. While the therapist’s empathy obviously influences her or his OT level, this increase appears to be accompanied by a reduction in the patient’s symptoms. Although no causality can be inferred here, it seems reasonable to assume that OT has an influence. Mu et al. (44), for example, found that the administration of OT not only increases behavioral synchrony in coordination games, but also promotes the synchronization of brain oscillations in the alpha band. Zilcha-Mano et al. (35) found that lower therapist-patient synchrony predicts changes in oxytocin levels during treatment, which in turn is associated with a smaller reduction in depressive symptoms during therapy.

**Figure 1:**
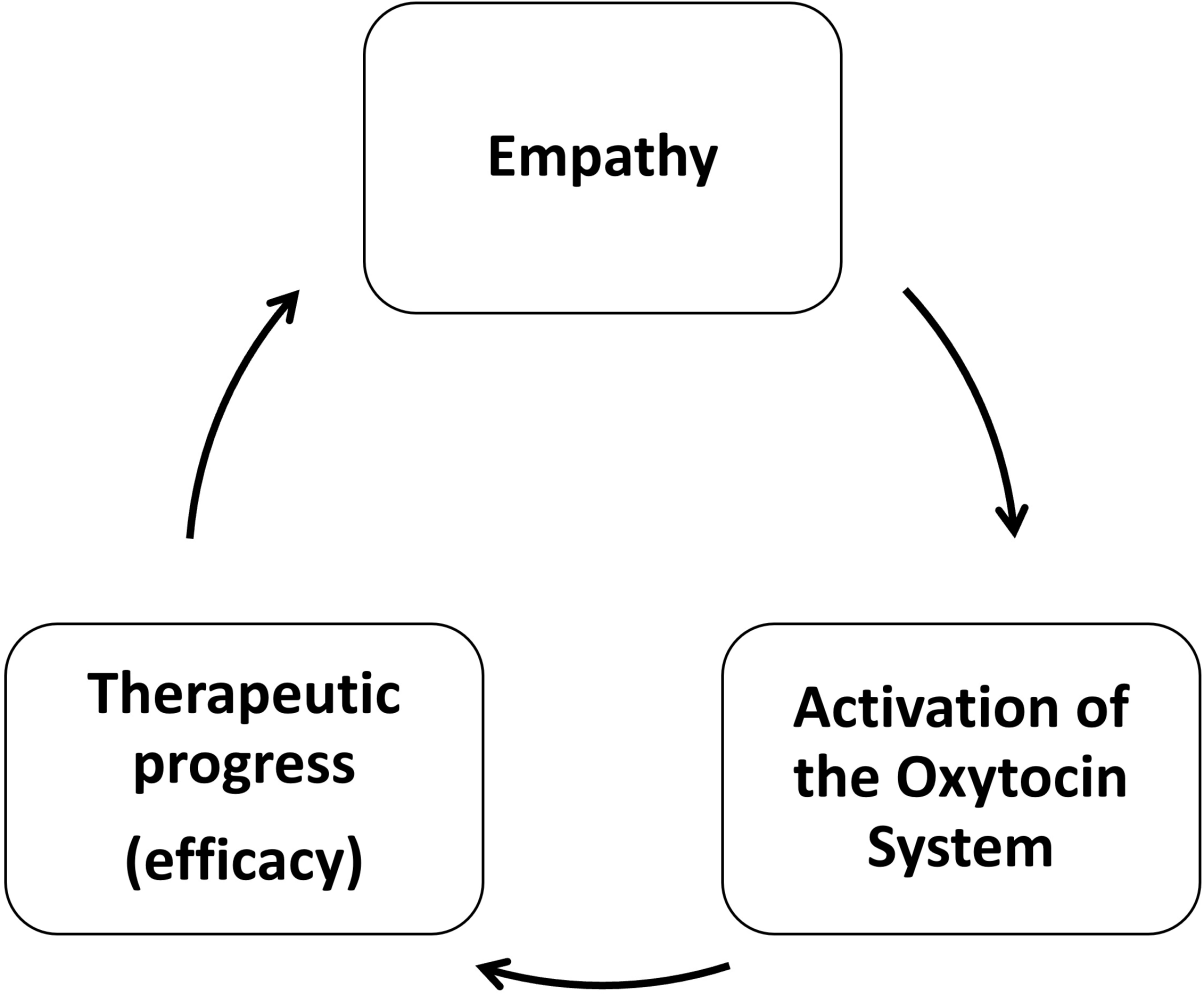
Self-reinforcing process of therapeutic alliance, OT activation and progress of psychotherapy.

While these findings support a model wherein empathy and OT form a self-reinforcing cycle that facilitates therapeutic progress (see **Figure 1**), several caveats apply. Measures of empathy vary widely across studies, complicating direct comparisons. Many studies rely on peripheral OT measures (e.g., plasma, saliva) or intranasal administration, which do not necessarily reflect central OT activity and differ in their ecological validity. Additionally, some designs lack appropriate control conditions, limiting causal conclusions. Individual differences, such as baseline attachment style or empathizing-systemizing cognitive profiles, further moderate these effects and deserve greater attention.

These limitations can additionally be applied to our next construct of social synchrony.

## Social synchrony

Social synchrony may underlie these OT-empathy links. Zilcha-Mano et al. (35) found that patient-therapist synchrony correlates with treatment success, with OT implicated as a mediating mechanism (6). Influs et al. (45, 46) implemented an eight-session intervention targeting synchrony and perspective-taking, reporting increases in both OT and empathy. Mimicry, a related synchronous behavior, is proposed to facilitate cognitive and emotional empathy (47). Reviews indicate that parent-infant synchrony correlates with parental OT levels (48), and experimental evidence shows that synchronous social interactions stimulate endogenous OT release in dyads (49). Moreover, oxytocin has been shown to enhance automatic imitation and facial mimicry, potentially mediating improved emotion recognition (50, 51).

As described in the before within the perspective of empathy, empirical evidence tentatively supports interaction effects in which social synchrony activates the OT system, which in turn enhances these social processes and therapeutic outcomes. However, more rigorous, longitudinal, and mechanistically informed research is needed to clarify causal pathways, measurement validity, and boundary conditions of this model.

## Fostering trust and cooperation

Trust and cooperation are fundamental components of psychotherapy that significantly influence the therapeutic alliance (52). Both constructs appear to be interactively linked with oxytocin (OT), though findings in this area are complex, sometimes contradictory, and moderated by multiple factors.

Several experimental studies have reported that intranasal OT administration can increase trust and cooperative behaviors. For example, Kosfeld et al. (53) found that OT enhanced trust in a financial investment game, and Declerck et al. (54) observed increased cooperation following OT administration. However, subsequent research has yielded mixed results. Declerck et al. (55) failed to replicate the trust-enhancing effect of OT in a similar paradigm, and Baumgartner et al. (56) showed that while OT prevented the typical decline in trust after betrayal, the overall effects on trust were nuanced. A critical review by Nave et al. (57) concluded that there is no consistent, robust evidence for a general effect of OT on human trust. Supporting this, Walum et al. (58) conducted a meta-analysis highlighting considerable variability in OT effects on social behavior, emphasizing the influence of context, individual differences, and methodological factors in explaining inconsistent findings.

These inconsistencies may partly stem from methodological variations, including differences in OT dosage, timing of administration, participant characteristics, and experimental tasks. Additionally, the nature of “trust” itself varies across studies—ranging from situational trust in economic games to dispositional or relational trust—limiting direct comparability.

Contextual and individual differences appear to moderate OT’s effects on trust and cooperation. For instance, Van IJzendoorn and Bakermans-Kranenburg (59) meta-analysis suggested that OT preferentially enhances trust toward ingroup members, emphasizing the social context. Conversely, Bartz et al. (60) found that OT reduced trust in individuals with borderline personality disorder, highlighting the importance of personal traits. Shou et al. (61) proposed that OT’s effects might reflect reduced caution or fear of betrayal rather than genuine increases in trust. Moreover, Mikolajczak et al. (62) emphasized the dependency of OT’s prosocial effects on situational factors. Regarding cooperation, social context and individual social value orientation—whether a person tends toward prosocial or self-oriented behavior—moderate OT’s impact, especially in economic game paradigms (54). Other moderators such as age and gender have also been reported (63, 64).

Endogenous OT levels have also been linked to trust-related behaviors. Zak et al. (65) found positive correlations between peripheral OT and both intentions to trust and trustworthy actions (reciprocation) in economic games. Similarly, Kiss et al. (66) reported increased OT during a trust-based secret-sharing task compared to control conditions. However, it is important to note that peripheral OT measures (blood or saliva) may not directly reflect central OT activity, and the exact relationship between peripheral and central OT remains unclear.

**Figure 2:**
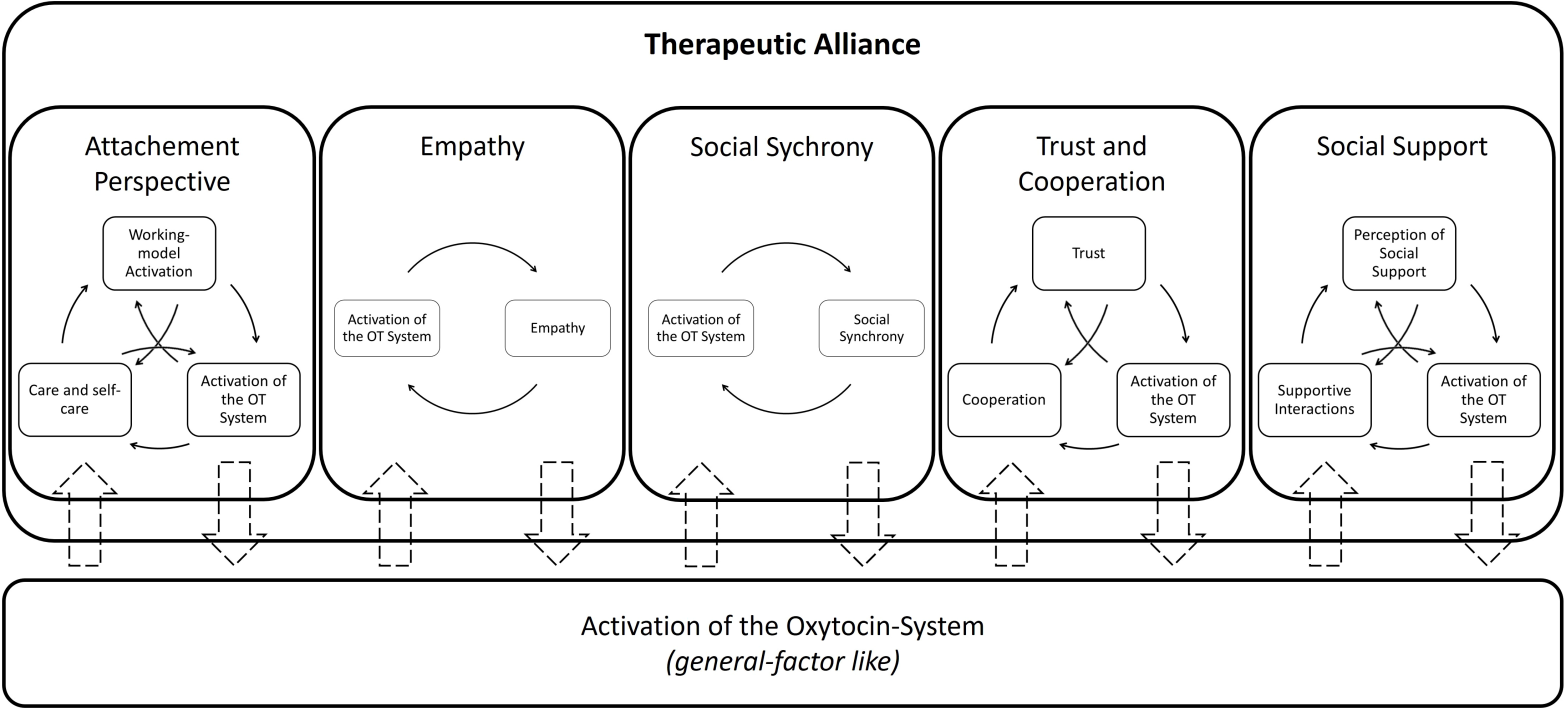
Integration of the single broaden-and-build-cycles of OT from a therapeutic alliance perspective.

Taken together, these findings suggest a complex, multidirectional relationship between trust, cooperation, and OT activity, strongly moderated by individual and contextual factors. We therefore propose a tentative self-reinforcing mechanism whereby therapeutic interventions that foster trust may elevate OT activity, which in turn facilitates trust-related cognitions and behaviors, further activating the OT system (see **Figure 2**). These self-reinforcing processes remain a theoretical model, requiring empirical validation, especially in clinical psychotherapy contexts.

## Social support

Social support is a psychological construct stemming from research on stress and health behaviors (67). Hence, it is considered a separate component in this mini review.

As early as the late 1990s, Carter (68) demonstrated that the accumulation of social support is associated with increased basal oxytocin responses to stress in animal models. Regarding the regulation of the hypothalamic-pituitary-adrenal (HPA) axis, oxytocin is hypothesized to mediate social buffering by attenuating physiological stress responses. In humans, Grewen et al. (69) found that self-reported partner support correlated with elevated plasma oxytocin levels following a warm contact intervention. Moreover, these authors suggested that partner support combined with increased oxytocin levels may have cardioprotective effects by modulating sympathetic nervous system activity and reducing blood pressure in women. However, it is important to note that plasma oxytocin levels represent peripheral measures and their relationship to central oxytocin activity remains indirect and not fully understood.

To further explore the interplay between social support, stress buffering, and oxytocin, Heinrichs et al. (70) conducted a placebo-controlled, double-blind study in humans. Participants received either intranasal oxytocin (24 IU) or placebo 50 minutes before exposure to the Trier Social Stress Test, combined with either social support or no support during the stressor. Results indicated an anxiolytic effect of oxytocin and, notably, the combination of oxytocin administration and social support was associated with the lowest cortisol responses and greater subjective calmness. These findings suggest that oxytocin may enhance the stress-buffering effects of social support, though further replication is needed to confirm these effects.

Neumann (71) discusses the mediating role of oxytocin in the positive effects of close social interactions on emotional well-being and health for many species, primarily based on rodent, sheep and primates’ studies. In these models, even subtle social stimuli activate oxytocin neurons. While such findings provide valuable insight into potential mechanisms, direct experimental evidence for similar oxytocinergic activation in the human brain following subtle social interactions—such as touch, hugging, or conversation — is currently lacking. Human research typically relies on peripheral oxytocin measurements in blood or saliva as indirect indicators of overall oxytocin system activation (72). The authors highlight that “activation of the brain OXT system even by subtle social stimuli is of particular relevance in the context of OT as a mediator of the positive effects of social support on stress responsiveness, as the OT system is closely linked to stress regulation” (72, p. 8), but emphasize the need for further research to substantiate these claims.

Consistent with the Tend-and-Befriend theory—which posits that social affiliation serves as a stress-coping mechanism, particularly in females—oxytocin responsiveness has been shown to predict the desire for social contact and support following social stress (73). Taken together, it is plausible that social stress induces support-seeking behavior, and that social support—especially emotional support—may alleviate psychological and physiological stress via oxytocinergic pathways. Nevertheless, no study to date has conclusively demonstrated direct activation of the central oxytocin system by social support in humans.

Despite these gaps, there is broad consensus that interventions aimed at enhancing social support positively impact health and well-being (74). It is hypothesized that oxytocin may partially mediate these beneficial effects (72), although this remains to be definitively established. Animal research supports the notion that partner support triggers oxytocin release and attenuates HPA axis responses to stress (75), but translating these findings to humans warrants cautious interpretation.

## Discussion

Based on previous research, this paper proposes five distinct oxytocin-related reciprocal self-reinforcing processes that may contribute to the development and strengthening of the therapeutic alliance. Each cycle outlines specific psychological factors that are hypothesized to activate the oxytocin system. In turn, activation of this system is assumed to reinforce these very factors, potentially creating a reciprocal dynamic that further promotes oxytocin release.

Taking a broader perspective, it appears plausible that activation of the oxytocin (OT) system does not influence only the specific psychological factor that initially triggered it but also exerts effects on other related factors. We therefore propose a more complex interplay between these five reciprocal self-reinforcing processes, as illustrated in **Figure 2**. For instance, empathy and synchrony may lead to increased trust and cooperation (76). Empirical findings also suggest a link between empathy and the therapeutic alliance (37). From an attachment theory perspective, both empathy and supportive behavior are considered fundamental to the development of attachment security. Additionally, research has shown that OT can simultaneously influence empathy, trust, and cooperation (77). These findings support our hypothesis that OT may function as a general facilitating factor, amplifying the effects of single psychological mechanisms and contributing to a broader, self-reinforcing dynamic beyond isolated reciprocal self-reinforcing processes.

## Theoretical and methodological considerations

While our model is informed by a growing body of evidence linking oxytocin (OT) to social bonding, stress regulation, and affiliative behaviors, we acknowledge that the current human literature remains heterogeneous, and in many areas, inconclusive. Notably, the association between classic attachment theory—primarily developed in the context of child–caregiver relationships—and the therapeutic alliance in psychotherapy is conceptually and empirically complex. Although both constructs involve interpersonal trust and perceived emotional safety, they emerge in distinct relational contexts and are typically assessed with different instruments. Current alliance measures (e.g., WAI) emphasize agreement on tasks and goals, and the perceived emotional bond, but do not directly map onto adult attachment styles such as avoidance or anxiety, which are usually measured in romantic or general interpersonal domains.

Moreover, while animal research robustly links oxytocin to attachment behaviors, direct evidence in humans remains limited. Studies suggest that early environmental factors—such as attachment experiences or early adversity—may moderate OT effects, rather than OT being a direct biological marker of secure attachment (22, 27). We therefore interpret the relationship between OT and attachment-related constructs with appropriate caution.

Similarly, although there are promising studies linking OT to aspects of the therapeutic process (e.g., 19, 35), the available evidence remains sparse, and findings are mixed. For instance, Ellenbogen et al. (19) showed that intranasal OT accelerated early alliance development in individuals with major depressive disorder, but this effect dissipated by mid-therapy as placebo recipients “caught up.” In other words, while OT may play a modulatory role in alliance formation, its therapeutic effects are likely time-sensitive and context-dependent.

Furthermore, methodological differences between studies make comparisons difficult. Endogenous OT levels measured in blood or saliva do not necessarily reflect central OT activity and may be influenced by numerous peripheral factors. Likewise, intranasal administration of synthetic OT introduces pharmacological effects that are not directly comparable to naturally occurring release. For this reason, we distinguish between findings based on (a) peripheral OT measurement, (b) intranasal OT administration, and (c) behavioral or psychological proxies of OT system activation (e.g., trust, synchrony, empathy).

While our broaden-and-build model posits reciprocal relationships between psychological processes and OT system activation, we fully recognize the preliminary nature of this proposal. The model is intended as a heuristic framework to stimulate further empirical research, not as a definitive account of OT function in psychotherapy. As such, we call for more rigorous, longitudinal, and mechanistically-informed studies to better understand the complex and likely bidirectional relationships between oxytocin, therapeutic processes, and clinical outcomes.

## Conclusion

Taking an even broader perspective, it becomes clear that many of the assumptions put forward in this paper are based on fragmented evidence. We also acknowledge that numerous pieces of the puzzle are still missing. Therefore, we have identified several research gaps that should be addressed in future studies. Closing these gaps is both important and worthwhile in order to gain a deeper understanding of how the therapeutic alliance may influence broaden-and-build cycles and, ultimately, the oxytocin system. Both the therapeutic alliance and oxytocin have been independently associated with symptom reduction and enhanced well-being. It is therefore conceivable that their effects are closely intertwined—perhaps even two sides of the same coin, or more precisely, reflecting a common underlying mechanism.
